# Supplementary material for: Neural networks can learn to utilize correlated auxiliary noise
Source: Sci Rep. 2021 Nov 3;11:21624. doi: 10.1038/s41598-021-00502-4 (PMC8566565; doi:10.1038/s41598-021-00502-4)
Supplement: Supplementary file 1 — Supplementary Information. [file 41598_2021_502_MOESM1_ESM.pdf]

Neural networks can learn to utilize correlated auxiliary noise

Aida Ahmadzadegan[1,2,3,*], Petar Simidzija[4], Ming Li[5], and Achim Kempf[1,3,6]

[1]Perimeter Institute for Theoretical Physics, Waterloo, Ontario, N2L 2Y5, Canada
[2]ForeQast Technologies Limited, Waterloo, Ontario, N2L 5M1, Canada
[3]Department of Applied Mathematics, University of Waterloo, Waterloo, Ontario, N2L 3G1, Canada
[4]Department of Physics and Astronomy, University of British Columbia, Vancouver, British Columbia, V6T 1Z4, Canada
[5]Cheriton School of Computer Science, University of Waterloo, Waterloo, Ontario, N2L 3G1, Canada
[6]Institute for Quantum Computing, University of Waterloo, Waterloo, Ontario, N2L 3G1, Canada
*ahmadzadegan.aida@gmail.com

Network architecture, training and evaluation details

- **Network architecture.** We build our baseline convolutional neural network model with 3 convolutional layers (Conv2D), 2 MaxPooling layers, 4 Dropout layers, and 2 fully-connected layers. Table 1 shows the network architecture used in our baseline network. This is a slightly modified version of the architecture used in[1]. We use L2 weight regularizer with a very small regularization hyperparameter $\alpha = 0.0005$ in the first convolutional layer to reduce the overfitting. All Conv2D layers and the first fully connected dense layer have ReLU[2] as their activation function and the last dense layer has softmax as its linear activation function.

- **Training and evaluation.** All networks were initialized using a default glorot uniform kernel initializer[3] and trained using the Adam optimizer with its default parameters[4]. We train the networks on the modified Fashion-MNIST training set for a specific number of epochs which is determined using the early stopping method to prevent overfitting. We chose a batch size of 32 and trained the network for 30 times and reported the average of the test accuracy and its standard deviation for each data point that is shown in Fig.3 of the paper. As was mentioned in Sec.3 of the paper, we create three types of data sets based on Fashion-MNIST grey scale images. For the type A data set, we add to the images a black bezel of 6 pixel width by zero-padding. For the type B data set, we add noise only to the image and leave the bezel clear, as, e.g., in Fig.2a in the paper, and for the type C data set, we add noise to both the image and the bezel, as, e.g., in Figs.2b-f in the paper.

  Let us look at the process of creating data set type B as an example. For the case where we want the images to consist of 50% noise, first, we rescale each MNIST image with original pixel values ranging between 0 and 255 and its add-on black bezel (pixel value zero) to an interval of [0.25, 0.75]. We rescale the images' pixel values so that when we add 50% of the noise which is originally chosen to have the amplitude interval [-0.5,0.5], the pixel values of the noisy image do not overflow 1 or underflow 0. Similarly, to mention another example, if we want to have 70% noise, we need to rescale original images and their bezel to an interval of [0.35,0.65]. Similarly, for other percentages of the noise, we do a corresponding rescaling, accordingly.

Highly complex noise correlations and quantum UCAN

Quantum machines may offer advantages for neural networks using UCAN, especially in the regime of highly complex noise, as a quantum machine with R qubits can store probability distributions described by a 2^R-dimensional Hilbert space. Also, while classically it is generally prohibitively expensive to draw from high-dimensional probability distributions, quantum machines allow one to easily draw from any of its states' probability distribution, through measurement. Further, the quantumness provides a source of true randomness rather than approximate randomness, as the violation of Bell inequalities shows, see, e.g.,[5,6]. In addition, the quantum mechanical Hilbert space of probability distributions is richer than that of classical distributions, due to the additional dependence on the choice of measured observables. This in turn allows entangled states that violate Bell-type inequalities to describe correlations, say between noise on data and auxiliary noise, that could not arise from localized classical dynamics.

As we show in the next section, the method for generating noise that we used in this paper can also be viewed as an accurate classical simulation of the quantum noise generated by a quantum system such as a quantum field in a suitable quantum state.

For example, the generating of low-complexity noise as a linear combination of Fourier sine functions with Gaussian distributed coefficients can be viewed as accurately simulating the vacuum fluctuations of a 2-dimensional neutral massless Klein-Gordon quantum field (which is similar to one polarization of the quantized electromagnetic field) discretized to a 40×40 grid and with an ultraviolet cutoff determined by the dimension of the noise space. On quantum field fluctuations, see, e.g.,[7–16]

The entanglement entropy in the vacuum state obeys an area law and is correspondingly low, consistent with the fact that the noise here is of low algorithmic complexity. For the relationship between Shannon entropy (here in the form of von Neumann entropy) and algorithmic complexity, see, e.g.,[17]. The statistics of the high complexity noise generated using white noise panels also matches the field's fluctuations, namely if the field's state is a suitable superposition of field-amplitude eigenstates.

While a quantum field can, therefore, generate the noise that we considered in our experiments, it is also capable to generate noise of extremely higher complexity. In fact, it is known[18], that any generic pure state is close to being maximally entangled and possesses close to maximum entanglement entropy between two equal size partitions of the system, such as here the bezel and the image. The almost maximal von Neumann entropies of the noise on the data and the auxiliary noise then imply a correspondingly almost maximal algorithmic complexity of the noise, illustrating the ability of quantum systems to efficiently store and draw from truly highly complex probability distributions.

Layer	Output shape	Function	Number of parameters
Conv2D_1	(None, 26, 26, 32)	Convolution 3×3	320
MaxPooling2D_1	(None, 13, 13, 32)	Maxpool 2×2	0
Dropout_1	(None, 13, 13, 32)	Dropout 0.25	0
Conv2D_2	(None, 11, 11, 64)	Convolution 3×3	18496
MaxPooling2D_2	(None, 5, 5, 64)	Maxpool 2×2	0
Dropout_2	(None, 5, 5, 64)	Dropout 0.25	0
Conv2D_3	(None, 3, 3, 128)	Convolution 3×3	73856
Dropout_3	(None, 3, 3, 128)	Dropout 0.4	0
Flatten_1	(None, 1152)	Reshape to a vector	0
Dense_1	(None, 128)	Fully connected layer	147584
Dropout_4	(None, 128)	Dropout 0.3	0
Dense_2	(None, 10)	Fully connected layer	1290

Table 1. Network architecture used in our experiments

Quantum fields as a source of noise

As we mentioned in Sections 3.2 and 4 of the paper, the low complexity noise that we used for our experiments is generated using sinusoidal noise panels with prefactors drawn from Gaussian probability distributions. We now show that this method of generating noise can be viewed as exactly simulating the quantum fluctuations of the amplitudes of a bandlimited massless neutral scalar field in the vacuum state.

Let $\hat{\phi}(\boldsymbol{x},t)$ denote a free, neutral, scalar quantum field in a flat spacetime of spatial dimension d, confined to a d-dimensional spatial hypercube of side length $L = 1$ with Dirichlet boundary conditions $\hat{\phi}(\boldsymbol{x},t)|_{\text{boundary}} = 0$. For generating 2-dimensional images, we choose the dimension to be $d = 2$. $\hat{\phi}(\boldsymbol{x},t)$ satisfies the Klein-Gordon equation

$$(\Box + m^2)\hat{\phi}(\boldsymbol{x},t) = 0, \tag{1}$$

the canonical commutation relations

$$\left[\hat{\phi}(\boldsymbol{x},t), \hat{\phi}(\boldsymbol{x}',t)\right] = \left[\hat{\pi}(\boldsymbol{x},t), \hat{\pi}(\boldsymbol{x}',t)\right] = 0, \quad \left[\hat{\phi}(\boldsymbol{x},t), \hat{\pi}(\boldsymbol{x}',t)\right] = \mathrm{i}\delta(\boldsymbol{x}-\boldsymbol{x}'), \tag{2}$$

and the self-adjointness condition $\hat{\phi}^\dagger(\boldsymbol{x},t) = \hat{\phi}(\boldsymbol{x},t)$, where $\hat{\pi}(\boldsymbol{x},t) := \partial_t \hat{\phi}(\boldsymbol{x},t)$ and m is the field's mass. For our simulations, we chose $m = 0$. We express the field in terms of the modes of its Fourier sine expansion

$$\hat{\phi}(\boldsymbol{x},t) = \sum_{\boldsymbol{n} \in \mathbb{N}^d} \hat{\phi}_{\boldsymbol{n}}(t) b_n(\boldsymbol{x}), \tag{3}$$

where the $b_n(\boldsymbol{x})$, with $\boldsymbol{n} = (n_1, \ldots, n_d) \in \mathbb{N}^d$, are given by

$$b_n(\boldsymbol{x}) = 2^{d/2} \prod_{i=1}^{d} \sin\left(\frac{n_i \pi x}{L}\right). \tag{4}$$

The mode operators $\hat{\phi}_{\boldsymbol{n}}(t)$ then read

$$\hat{\phi}_{\boldsymbol{n}}(t) = \frac{1}{\sqrt{2\omega_{\boldsymbol{n}}}}\left(e^{\mathrm{i}\omega_{\boldsymbol{n}}t}\hat{a}^{\dagger}_{\boldsymbol{n}} + e^{-\mathrm{i}\omega_{\boldsymbol{n}}t}\hat{a}_{\boldsymbol{n}}\right). \tag{5}$$

and we have, similarly, for their canonically conjugate operators, $\hat{\pi}_{\boldsymbol{n}}(t)$:

$$\hat{\pi}_{\boldsymbol{n}}(t) = \mathrm{i}\sqrt{\frac{\omega_{\boldsymbol{n}}}{2}}\left(e^{\mathrm{i}\omega_{\boldsymbol{n}}t}\hat{a}^{\dagger}_{\boldsymbol{n}} - e^{-\mathrm{i}\omega_{\boldsymbol{n}}t}\hat{a}_{\boldsymbol{n}}\right). \tag{6}$$

The frequency reads $\omega_{\boldsymbol{n}} = \sqrt{|\boldsymbol{k}_{\boldsymbol{n}}|^2 + m^2}$, and the wave vectors $\boldsymbol{k}_{\boldsymbol{n}}$ are defined as $\boldsymbol{k}_{\boldsymbol{n}} := \frac{\pi}{L}(n_1, n_2, \ldots, n_d)$. The annihilation and creation operators $\hat{a}_{\boldsymbol{n}}$ and $\hat{a}^{\dagger}_{\boldsymbol{n}}$ obey

$$[\hat{a}_{\boldsymbol{n}}, \hat{a}_{\boldsymbol{m}}] = [\hat{a}^{\dagger}_{\boldsymbol{n}}, \hat{a}^{\dagger}_{\boldsymbol{m}}] = 0, \quad [\hat{a}_{\boldsymbol{n}}, \hat{a}^{\dagger}_{\boldsymbol{m}}] = \delta_{\boldsymbol{n},\boldsymbol{m}}, \tag{7}$$

and the vacuum state $|0\rangle$ obeys $\hat{a}_{\boldsymbol{n}}|0\rangle = 0$ for all n. In addition to the infrared regularization provided by the box, we also impose an ultraviolet regularization that bandlimits the field by truncating the sum in Eq.(3). In our simulations, we have $d = 2$ and we truncated the sum at $n_i = 5, 15, 22$, leading to $5^2, 15^2$ and 22^2 dimensional noise spaces, respectively.

Let us now derive the probability distribution for field amplitude measurements in the vacuum state $|0\rangle$. To this end, we utilize that, in Eq.3, the field is decomposed into independent harmonic oscillators. The pairs of conjugate degrees of freedom $\hat{\phi}_{\boldsymbol{n}}(t)$ and $\hat{\pi}_{\boldsymbol{n}}(t)$ are self-adjoint and satisfy $[\hat{\phi}_{\boldsymbol{n}}(t), \hat{\pi}_{\boldsymbol{n}'}(t)] = \mathrm{i}\delta_{\boldsymbol{n},\boldsymbol{n}'}$. We choose a fixed time, such as $t = 0$. In the $\hat{\phi}_{\boldsymbol{n}}$ eigenbasis, we have $\hat{\phi}_{\boldsymbol{n}} = \phi_{\boldsymbol{n}}$ and $\hat{\pi}_{\boldsymbol{n}} = -\mathrm{i}\frac{\partial}{\partial \phi_{\boldsymbol{n}}}$, so that the condition $\hat{a}_{\boldsymbol{n}}|0\rangle = 0$ becomes

$$\sqrt{\frac{\omega_{\boldsymbol{n}}}{2}}\left[\phi_{\boldsymbol{n}} + \frac{\mathrm{i}}{\omega_{\boldsymbol{n}}}\left(-\mathrm{i}\frac{\partial}{\partial \phi_{\boldsymbol{n}}}\right)\right]\psi_0(\phi_{\boldsymbol{n}}) = 0, \tag{8}$$

where we have one equation for each value of $\boldsymbol{n}$. These equations are solved to yield the normalized wavefunctions of the ground states of harmonic oscillators,

$$\psi_0(\phi_{\boldsymbol{n}}) = \left(\frac{\omega_{\boldsymbol{n}}}{\pi}\right)^{1/4}\exp\left(-\frac{\omega_{\boldsymbol{n}}}{2}\phi^2_{\boldsymbol{n}}\right). \tag{9}$$

Thus when the quantum field is in its ground state, $|0\rangle$, measurement outcomes of the amplitudes of each of the field modes $\hat{\phi}_{\boldsymbol{n}}$ are each independently following a Gaussian probability distribution function $|\psi_0(\phi_{\boldsymbol{n}})|^2$ with mean zero and variance $1/2\omega_{\boldsymbol{n}}$. By drawing from this probability distribution and using Eq.(3), we obtain a sample measurement of the field. This matches exactly the calculations of the low-complexity noise panels in our experiments.

References

1. Le, J. Fashion-MNIST. https://github.com/khanhnamle1994/fashion-mnist/commits?author=khanhnamle1994 (2018).
2. Maas, A. L., Hannun, A. Y. & Ng, A. Y. Rectifier nonlinearities improve neural network acoustic models. In *International Conference on Machine Learning (ICML)* (2013).
3. Glorot, X. & Bengio, Y. Understanding the difficulty of training deep feedforward neural networks. In *Proceedings of the Thirteenth International Conference on Artificial Intelligence and Statistics*, 249–256 (2010).
4. Kingma, D. P. & Ba, J. Adam: A method for stochastic optimization. *arXiv:1412.6980* (2014).
5. Weihs, G., Jennewein, T., Simon, C., Weinfurter, H. & Zeilinger, A. Violation of Bell's inequality under strict Einstein locality conditions. *Phys. Rev. Lett.* **81**, 5039–5043, DOI: 10.1103/PhysRevLett.81.5039 (1998). quant-ph/9810080.
6. Rosenfeld, W. *et al.* Event-ready bell test using entangled atoms simultaneously closing detection and locality loopholes. *Phys. Rev. Lett.* **119**, 010402, DOI: 10.1103/PhysRevLett.119.010402 (2017).
7. Liddle, A. R. & Lyth, D. H. *Cosmological inflation and large-scale structure* (Cambridge university press, 2000).
8. Mukhanov, V. *Physical foundations of cosmology* (Cambridge university press, 2005).
9. Mukhanov, V. & Winitzki, S. *Introduction to quantum effects in gravity* (Cambridge university press, 2007).
10. Birrell, N. D. & Davies, P. C. W. *Quantum fields in curved space*. Cambridge Monographs on Mathematical Physics (Cambridge University Press, 1982).

11. Kempf, A., Mangano, G. & Mann, R. B. Hilbert space representation of the minimal length uncertainty relation. *Phys. Rev. D* **52**, 1108–1118, DOI: 10.1103/PhysRevD.52.1108 (1995).
12. Kempf, A. Fields over unsharp coordinates. *Phys. Rev. Lett.* **85**, 2873–2876, DOI: 10.1103/PhysRevLett.85.2873 (2000).
13. Chatwin-Davies, A., Kempf, A. & Martin, R. T. W. Natural covariant planck scale cutoffs and the cosmic microwave background spectrum. *Phys. Rev. Lett.* **119**, 031301, DOI: 10.1103/PhysRevLett.119.031301 (2017).
14. Kempf, A., Chatwin-Davies, A. & Martin, R. T. W. A fully covariant information-theoretic ultraviolet cutoff for scalar fields in expanding friedmann robertson walker spacetimes. *J. Math. Phys.* **54**, 022301, DOI: 10.1063/1.4790482 (2013). https://doi.org/10.1063/1.4790482.
15. Kempf, A. Covariant information-density cutoff in curved space-time. *Phys. Rev. Lett.* **92**, 221301 (2004).
16. Pye, J., Donnelly, W. & Kempf, A. Locality and entanglement in bandlimited quantum field theory. *Phys. Rev. D* **92**, 105022, DOI: 10.1103/PhysRevD.92.105022 (2015).
17. Li, M. & Vitányi, P. *An introduction to Kolmogorov complexity and its applications*. Texts in Computer Science (Springer International Publishing, 2019), 4th edn.
18. Page, D. N. Information in black hole radiation. *Phys. Rev. Lett.* **71**, 3743–3746, DOI: 10.1103/PhysRevLett.71.3743 (1993).
